# Associations of Bioelectrical Impedance-Derived Phase Angle and Hydration Parameters with Clinical Severity in Ambulatory Chronic Heart Failure

**DOI:** 10.3390/jcm15062315

**Published:** 2026-03-18

**Authors:** Carolina Moreno-Torres-Taboada, Francisco José Sánchez-Torralvo, María García-Olivares, Sonia Castillo-López, Alejandro Pérez-Espejo, José María Pérez-Ruiz, Gabriel Olveira

**Affiliations:** 1 Endocrinology and Nutrition Department, Hospital Regional Universitario de Málaga, 29010 Málaga, Spain; carolina.mtaboada@gmail.com (C.M.-T.-T.); merygarcia96@gmail.com (M.G.-O.); 2 Department of Medicine and Dermatology, Faculty of Medicine, University of Malaga, 29016 Málaga, Spain; alepex2706@gmail.com; 3 Instituto de Investigación Biomédica de Málaga (IBIMA)—BIONAND Platform, 29590 Málaga, Spain; 4 Endocrinology and Nutrition Department, Hospital QuirónSalud, 29004 Málaga, Spain; 5 Cardiology Department, Hospital Regional Universitario de Málaga, 29010 Málaga, Spain; soniacl@gmail.com (S.C.-L.); chemanyi0869@gmail.com (J.M.P.-R.); 6 Biomedical Research Networking Center in Diabetes and Associated Metabolic Disorders (CIBERDEM), Instituto de Salud Carlos III, 29010 Málaga, Spain

**Keywords:** malnutrition, heart failure, bioelectrical impedance analysis, phase angle, hydration

## Abstract

**Background/Objectives:** Malnutrition and altered body composition are frequent in chronic heart failure (HF) and are associated with worse functional status and prognosis. Bioelectrical impedance analysis (BIA) is increasingly used in nutritional assessment, although its interpretation may be confounded by fluid overload. This study aimed to evaluate the association between BIA-derived parameters and clinical and biochemical markers of disease severity in ambulatory patients with chronic heart failure. **Methods:** This cross-sectional study included adult outpatients with chronic HF consecutively assessed in a specialised HF unit. Nutritional evaluation comprised anthropometry, handgrip strength, rectus femoris muscle ultrasound and BIA. Phase angle (PA) and hydration-related parameters were analysed in relation to New York Heart Association (NYHA) functional class and N-terminal pro-B-type natriuretic peptide (NT-proBNP) levels. Multivariable regression models adjusted for relevant clinical and BIA variables were applied. **Results:** A total of 115 patients were included (mean age 68.2 ± 12.6 years; 71.3% men). Mean PA was 4.6 ± 1.1°. Lower PA was independently associated with greater dyspnoea severity (*p* = 0.026) and higher NT-proBNP concentrations (*p* = 0.014). Higher total body water was positively associated with symptom burden (*p* = 0.013) and NT-proBNP levels (*p* < 0.001) and showed good discriminatory performance for identifying patients in the highest NT-proBNP quartile. **Conclusions:** In ambulatory patients with chronic HF, BIA-derived parameters reflecting cellular integrity and hydration status are independently associated with clinical and biochemical markers of disease severity. BIA may provide complementary information in nutritional assessment, although hydration-related confounding should be carefully considered. Future longitudinal studies should determine whether these bioimpedance-derived parameters can improve risk stratification and nutritional assessment in chronic heart failure.

## 1. Introduction

Heart failure (HF) is a complex clinical syndrome characterised by typical symptoms such as dyspnea, fatigue, and fluid retention, together with objective signs of cardiac dysfunction. It affects approximately 1–2% of the adult population and represents a major public health problem due to its high rates of hospitalisation, mortality, and healthcare expenditure [1]. Beyond its haemodynamic and neurohormonal consequences, HF is increasingly recognised as a systemic condition with profound metabolic and nutritional implications [1].

Malnutrition and loss of skeletal muscle mass are frequent in patients with HF and are consistently associated with worse functional capacity, increased complications, and poorer prognosis [2,3]. Chronic HF is often accompanied by systemic venous congestion, inflammation, and neurohormonal activation, which may promote intestinal oedema, anorexia, malabsorption, and catabolic imbalance, ultimately leading to malnutrition and cardiac cachexia [4,5]. The reported prevalence of malnutrition in HF varies widely, ranging from 25% to 60%, depending on the population studied, clinical setting, HF severity, and, importantly, the nutritional assessment method applied [6,7,8]. Regardless of the tool used, malnutrition has repeatedly been identified as an independent predictor of in-hospital mortality, rehospitalisation, and long-term adverse outcomes in HF patients [8,9,10].

In recent years, the Global Leadership Initiative on Malnutrition (GLIM) criteria have been proposed as a consensual framework for the diagnosis of malnutrition, introducing the mandatory assessment of skeletal muscle mass as a key phenotypic criterion [11]. Several techniques have been suggested for muscle mass evaluation, including dual-energy X-ray absorptiometry (DXA), computed tomography, and bioelectrical impedance analysis (BIA). This flexibility, while pragmatic, has also introduced heterogeneity in clinical practice and research, particularly in conditions characterised by fluid imbalance.

BIA has gained considerable attention as a non-invasive, rapid and bedside-accessible technique for the assessment of body composition. By measuring the electrical properties of body tissues, BIA provides estimates of fat mass, fat-free mass, total body water and derived parameters such as phase angle (PA) [12,13,14,15]. In several chronic conditions, including chronic kidney disease, dialysis-dependent patients and oncological populations, BIA-derived indices have demonstrated associations with functional status, nutritional risk and prognosis [16,17,18,19,20,21,22,23]. In these settings, PA in particular has emerged as a robust marker of cellular integrity and clinical outcomes.

In heart failure, BIA has been increasingly explored not only as a nutritional assessment tool but also as a means of evaluating congestion and fluid distribution [24,25]. Previous studies, largely conducted in acute or decompensated HF, have shown that BIA parameters and PA correlate with biomarkers of congestion, clinical oedema and changes in volume status during hospitalisation [26,27,28,29]. Moreover, lower PA values have been consistently associated with worse prognosis and higher mortality in heart failure populations. These findings support the biological plausibility of BIA as a clinically meaningful tool in this disease.

Nevertheless, the interpretation of BIA in chronic HF remains challenging. Electrical impedance is strongly influenced by hydration status, and extracellular fluid expansion may confound estimates of lean mass and muscle reserves [30]. As a consequence, it is unclear to what extent BIA-derived parameters in stable, chronic HF reflect true alterations in nutritional and muscular status, as opposed to the effects of persistent or subclinical overhydration. This uncertainty is particularly relevant when BIA is used to operationalise GLIM criteria, where misclassification of muscle mass could directly affect the diagnosis of malnutrition.

Despite the growing body of literature on BIA in heart failure, data specifically addressing its relationship with clinical and functional impairment in chronic, predominantly ambulatory patients remain limited. Most available studies have been conducted in hospitalised or acutely decompensated heart failure cohorts, where rapid changes in fluid status may strongly influence bioimpedance-derived measurements. In contrast, the potential role of BIA parameters in the evaluation of disease severity and nutritional status in stable ambulatory patients has been less extensively explored. This question is particularly relevant in the context of nutritional assessment, where bioimpedance-derived muscle indices are increasingly used to operationalise diagnostic criteria for malnutrition.

Therefore, the aim of the present study was to evaluate the association between BIA-derived parameters, particularly those reflecting hydration status and cellular integrity, and clinical and biochemical markers of disease severity in ambulatory patients with chronic HF in a stable outpatient population.

## 2. Materials and Methods

### 2.1. Study Design and Setting

This was a cross-sectional study conducted under routine clinical practice conditions. The study was carried out at the Heart Failure Unit of the Department of Cardiology and the Clinical Nutrition and Dietetics Unit of the Hospital Regional Universitario of Málaga.

Adult outpatients with a confirmed diagnosis of chronic heart failure (HF) who were routinely followed at the specialised Heart Failure Unit were consecutively assessed. The primary objective was to examine the association between bioelectrical impedance analysis (BIA)-derived parameters and clinical and biochemical markers of disease severity at baseline.

This study protocol was approved by the Provincial Research Ethics Committee of Málaga (protocol code #15122022; approval date 15 December 2022), and all participants provided written informed consent prior to inclusion. All procedures were conducted in accordance with the Declaration of Helsinki and local data protection regulations. The study protocol is available upon reasonable request.

### 2.2. Study Population

Eligible participants were adult ambulatory patients with a diagnosis of heart failure who were being followed at the specialised Heart Failure Unit.

Inclusion criteria were as follow:

(i) Age ≥ 18 years;
(ii) Confirmed diagnosis of chronic heart failure;
(iii) Outpatient follow-up at the Heart Failure Unit.

Exclusion criteria were as follows:

(i) Refusal or inability to provide informed consent;
(ii) Hospital admission within the preceding 30 days.

### 2.3. Study Variables and Nutritional Assessment

All data were collected using standardised case report forms and subsequently entered into a dedicated study database. The variables collected are detailed below.

#### 2.3.1. Clinical and Cardiological Variables

Demographic data included age and sex. Relevant comorbidities, including previously diagnosed diabetes mellitus and chronic medical treatments, were recorded.

Cardiological variables comprised left ventricular ejection fraction, aetiology of heart failure, heart failure phenotype (reduced or preserved ejection fraction), New York Heart Association (NYHA) functional class, and American Heart Association (AHA) heart failure stage.

#### 2.3.2. Anthropometric and Nutritional Variables

Body weight was recorded as current weight, usual weight, and adjusted weight when clinically indicated (e.g., obesity or presence of oedema). Height was measured using a stadiometer when feasible or self-reported otherwise. Body mass index (BMI) was calculated as weight divided by height squared (kg/m^2^) and interpreted according to ESPEN criteria.

Malnutrition was assessed according to the Global Leadership Initiative on Malnutrition (GLIM) framework, focusing exclusively on phenotypic criteria, assuming fulfilment of the etiologic criterion due to chronic disease, although inflammatory burden may differ among ambulatory patients. The phenotypic criteria evaluated included low BMI, unintentional weight loss, and reduced muscle mass.

Low BMI was defined using age-specific cut-offs in accordance with ESPEN recommendations, with thresholds of <20 kg/m^2^ for patients younger than 70 years and <22 kg/m^2^ for those aged 70 years or older. Unintentional weight loss was considered clinically relevant when exceeding 5% of usual body weight within the previous 6–12 months, in line with GLIM criteria [11,31].

Muscle ultrasound and handgrip strength variables were analysed exclusively for convergent validity with bioimpedance-derived parameters. As these measures were not study outcomes and reference values are being investigated separately, descriptive statistics are not reported.

#### 2.3.3. Bioelectrical Impedance Analysis

BIA was performed using a phase-sensitive, single-frequency (50 kHz) impedance analyser (AKERN, Florence, Italy) with a tetrapolar electrode configuration delivering an 800 μA current. Measurements were obtained with participants in the supine position after a minimum rest period of 5 min to ensure fluid redistribution. Measurements were performed under routine clinical practice conditions. Participants were instructed to remain in the supine position during the assessment, although fasting status, recent physical activity and diuretic intake were not systematically standardised.

Electrodes were placed on the right hand and foot following standardised procedures. The device was calibrated daily using the manufacturer-provided control circuit with known resistance and reactance values.

The parameters recorded included resistance and reactance standardised for height, phase angle (PA), standardised PA, body cell mass and its index, fat mass and fat mass index, fat-free mass index, appendicular skeletal muscle mass, skeletal muscle index, total body water, extracellular and intracellular water, and hydration status. PA was calculated as the arctangent of reactance divided by resistance and expressed in degrees. Standardised phase angle was derived using age- and sex-specific reference values from healthy populations.

Reduced muscle mass was defined using BIA-derived indices, including fat-free mass index (FFMI) and appendicular skeletal muscle mass index (ASMMI). Sex-specific cut-off values published in the GLIM consensus and ESPEN guidelines were applied to classify low muscle mass [11,32].

### 2.4. Laboratory Parameters

Routine laboratory data were collected, including haemoglobin, lymphocyte count, total protein, albumin, prealbumin, C-reactive protein, glucose, glycated haemoglobin, total cholesterol, triglycerides, vitamin D, and N-terminal pro-brain natriuretic peptide (NT-proBNP). Laboratory measurements were performed at the hospital’s central laboratory using standardised automated methods according to routine clinical protocols.

### 2.5. Nutritional Intervention

Information regarding nutritional intervention prior to and following the nutritional assessment was recorded. Interventions included dietary counselling, oral nutritional supplementation, enteral nutrition, or parenteral nutrition, as clinically indicated and according to standard clinical practice. These interventions were prescribed individually by the Clinical Nutrition team according to routine clinical judgement, based on the results of the nutritional assessment, oral intake, anthropometric findings and the overall clinical condition of the patient.

### 2.6. Statistical Analysis

Continuous variables are presented as mean ± standard deviation, and categorical variables as absolute and relative frequencies. Normality of continuous variables was assessed using graphical methods and the Shapiro–Wilk test, as appropriate.

Between-group comparisons were performed using Student’s *t* test or the Mann–Whitney U test according to data distribution. Pearson correlation coefficients were calculated to assess linear associations between phase angle and other body composition, anthropometric, functional and ultrasound-derived parameters.

The association between BIA parameters and dyspnoea severity (NYHA I–IV) was evaluated using ordinal logistic regression models. Results are reported as odds ratios (ORs) with 95% confidence intervals (CIs). Model fit was assessed using likelihood ratio tests and McFadden’s pseudo-R^2^.

Associations between BIA parameters and NT-proBNP levels were analysed using multivariable linear regression models with log-transformed NT-proBNP as the dependent variable. Model assumptions were verified by inspection of residual plots, and multicollinearity was assessed using variance inflation factors.

Variables included in multivariable models were selected a priori based on clinical relevance and previously reported associations with heart failure severity. These variables included age, sex, body mass index, heart failure phenotype and left ventricular ejection fraction.

To identify a high NT-proBNP phenotype, defined as values in the fourth quartile of the cohort distribution, binary logistic regression analyses were performed. Discriminatory performance was evaluated using receiver operating characteristic (ROC) curve analysis, and results are reported as area under the curve (AUC) values.

All statistical tests were two-sided, and a *p* value < 0.05 was considered statistically significant. All analyses were conducted using JAMOVI software (version 2.3.22 for Windows).

Sample size considerations: As this was an exploratory cross-sectional study conducted under routine clinical practice conditions, no a priori sample size calculation was performed. With a final sample of 115 patients and assuming α = 0.05, the available sample provided >80% power to detect standardised regression coefficients of approximately 0.25 in multivariable linear models including up to six predictors. This corresponds to small-to-moderate associations according to conventional effect size benchmarks.

### 2.7. Use of Generative Artificial Intelligence

Generative artificial intelligence tools were used exclusively for linguistic editing and improvement of grammar and style. No AI tools were used for study design, data collection, data analysis, or interpretation.

## 3. Results

### 3.1. Baseline Characteristics of the Study Population

A total of 115 patients with chronic heart failure (HF) were included in the analysis. The mean age was 68.2 ± 12.6 years, and 71.3% of participants were men. Previous diabetes mellitus was present in 30.4% of the cohort. According to the American Heart Association (AHA) classification, 56.5% of patients were classified as stage B and 43.5% as stage C heart failure.

Regarding symptom burden, 37.4% of patients were classified as New York Heart Association (NYHA) I, 44.3% as NYHA II, 15.7% as NYHA III and 2.6% as NYHA IV. Mean left ventricular ejection fraction was 38.4 ± 10.8%, and mean NT-proBNP concentration was 3171 ± 5727 pg/mL, as shown in Table 1.

Body composition assessment by BIA revealed a mean phase angle (PA) of 4.6 ± 1.1°. Hydration-related parameters indicated a mean total body water (TBW) of 41.4 ± 8.8 L and extracellular water (ECW) of 21.6 ± 5.2%. Relevant sex-related differences were observed for several body composition variables, including resistance, reactance, total body water, extracellular water and fat-free mass. BIA-derived parameters are detailed in Table 2.

The prevalence of Global Leadership Initiative on Malnutrition (GLIM) phenotypic criteria in the study population is summarised in Table 2. Using age-specific BMI cut-offs, low BMI was infrequent, whereas a relevant proportion of patients fulfilled criteria for recent weight loss. When muscle mass was assessed by BIA, the prevalence of low muscle mass varied depending on the index used, with differences observed between FFMI and ASMMI. Theoretically, all patients fulfilled the GLIM aetiological criterion due to chronic disease-related inflammation; however, it should be acknowledged that not all ambulatory heart failure patients present the same degree of inflammation or congestion. Taking this into account, the overall prevalence of malnutrition in this sample, as defined by the applied GLIM criteria, was 39.5%.

Phase angle showed moderate correlations with muscle ultrasound and handgrip variables (Supplementary Table S1), supporting the convergent validity of bioimpedance-derived cellular integrity parameters and their relationship with structural and functional muscle measures.

### 3.2. Association Between BIA Parameters and Dyspnoea Severity

**Figure 1:**
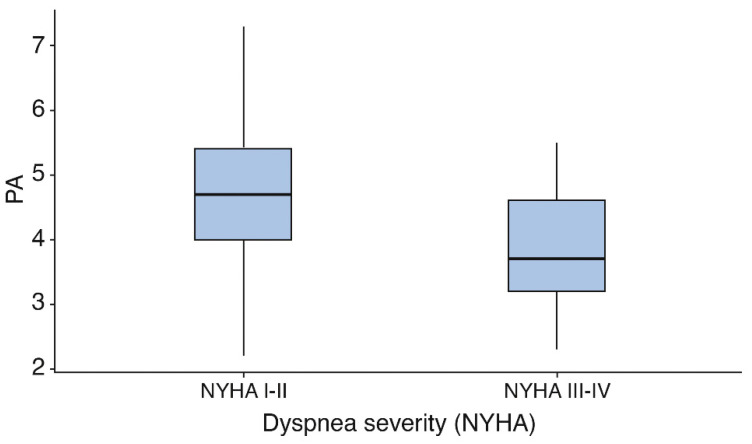
Phase angle (°) according to dyspnoea severity Boxplots represent median and interquartile range, with individual data points shown.

PA values progressively decreased with increasing dyspnoea severity. Patients classified as NYHA III–IV exhibited significantly lower PA values compared with those in NYHA I–II (Figure 1).

In multivariable ordinal logistic regression analysis, lower PA and higher total body water were independently associated with greater dyspnoea severity. Each 1° increase in PA was associated with 42% lower odds of being in a higher NYHA class, whereas total body water showed a positive association with symptom burden. Age was also independently associated with dyspnoea severity, while sex, body mass index, heart failure type and left ventricular ejection fraction were not significantly associated. These associations are detailed in Table 3.

### 3.3. Association Between BIA Parameters and N-Terminal Pro-B-Type Natriuretic Peptide (NT-proBNP) Levels

In multivariable linear regression analysis, lower phase angle and higher total body water percentage were independently associated with higher NT-proBNP levels after adjustment for age, sex, body mass index, heart failure type and left ventricular ejection fraction.

Left ventricular ejection fraction also showed a significant inverse association with NT-proBNP concentrations. The final model explained 41.5% of the variability in log-transformed NT-proBNP levels, with regression coefficients for each parameter shown in Table 4. Model diagnostics confirmed normal distribution of residuals and absence of multicollinearity.

### 3.4. Identification of a High NT-proBNP Phenotype

Given the absence of a universally accepted NT-proBNP threshold in this clinical context, high NT-proBNP was defined as values in the fourth quartile of the cohort distribution, a pragmatic approach frequently used in exploratory analyses to identify patients with relatively higher biomarker levels within a heterogeneous ambulatory population. Binary logistic regression analyses were performed to evaluate associations between bioelectrical impedance-derived parameters and membership in this high NT-proBNP group.

**Figure 2:**
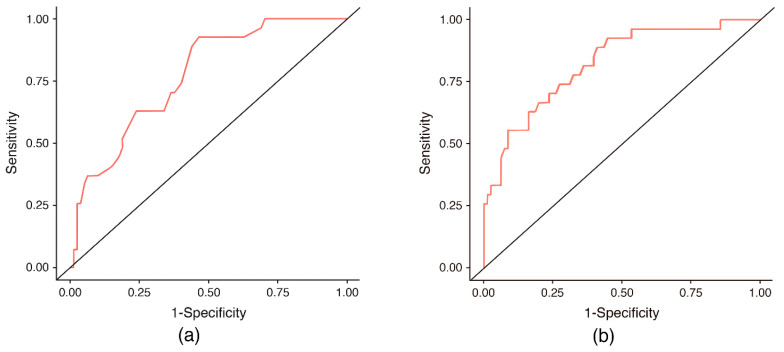
Receiver operating characteristic curves illustrating the ability of PA and TBW percentage to identify patients in the highest NT-proBNP quartile. (**a**) Phase angle (°) (**b**) Total body water (%).

In these models, both PA and TBW percentage were significantly associated with elevated NT-proBNP levels. Receiver operating characteristic (ROC) curve analysis was performed using the predicted probabilities derived from the binary logistic regression models built separately for PA and TBW. The optimal probability threshold for discrimination was selected using the Youden index. For PA, the highest Youden index showed a sensitivity of 92.6% and a specificity of 53.7% (AUC 0.77), while for TBW the highest Youden index showed a sensitivity of 74.1% and a specificity of 72.8% (AUC 0.82) (Table 5, Figure 2).

## 4. Discussion

### 4.1. Interpretation of Direct and Derived BIA Parameters in Chronic HF

Interpretation of BIA-derived body composition measures in patients with HF must account for the pathophysiological context of fluid imbalance. While PA, resistance and reactance are directly measured by BIA and reflect intrinsic electrical properties of tissues, derived estimates such as skeletal muscle mass or fat-free mass are generated through regression equations and classification algorithms that assume normative hydration states. In HF, where extracellular fluid expansion is common, these assumptions are violated, leading to systematic overestimation of muscle mass and underestimation of tissue depletion. Consequently, interpretation of BIA-derived muscle indices as robust nutritional markers is problematic in this population.

In addition, the use of single-frequency bioelectrical impedance analysis represents a methodological limitation in patients with altered fluid distribution. At a frequency of 50 kHz, impedance measurements may be particularly influenced by extracellular fluid expansion, which is common in heart failure and may affect the accuracy of algorithm-derived body composition estimates. In contrast, directly measured electrical parameters such as resistance, reactance and phase angle are less dependent on these predictive equations and may therefore provide more physiologically meaningful information in this clinical context.

In our cohort, malnutrition prevalence according to conventional BIA-based GLIM criteria was 39.5%, which was lower than the prevalence reported in heart failure cohorts assessed using established clinical or composite nutritional screening tools, ranging from 42.4% to 57% [4,7]. Compared with these reports, the low prevalence of malnutrition detected by conventional BIA metrics in our cohort suggests that algorithm-derived muscle indices may lack sensitivity in the setting of fluid overload. Therefore, integration of direct bioelectrical parameters (PA, resistance, reactance) and clinical context should be prioritised when assessing nutritional status in this patient group.

### 4.2. Associations of Phase Angle and Hydration with Disease Severity

PA has been widely proposed as an integrative marker of cellular health, reflecting the balance between cell membrane integrity and body cell mass [12,13,14,15,18]. In the present study, lower PA values were associated with greater symptom severity and higher NT-proBNP levels, reinforcing the concept that cellular compromise and muscle quality deterioration are closely linked to functional limitation in chronic HF. Previous studies have reported similar associations between low PA and adverse outcomes in HF populations, including increased mortality and reduced physical performance [25,33,34,35,36,37,38]. However, some of these investigations were conducted in hospitalised or acutely decompensated patients, where rapid shifts in fluid status and inflammatory activity may exert a stronger influence on BIA-derived measurements [9,26,28,29,39]. In contrast, our cohort consisted predominantly of stable, ambulatory patients with chronic disease, suggesting that the observed associations are not merely epiphenomena of acute congestion but may reflect more sustained alterations in body composition and cellular function.

Alternative techniques such as dual-energy X-ray absorptiometry and muscle ultrasound have also been proposed for the assessment of body composition and muscle mass in patients with heart failure. While DXA provides relatively accurate estimates of lean mass, its routine use may be limited by cost and accessibility. Muscle ultrasound offers a promising bedside method to evaluate muscle quantity and architecture, although standardisation and reference values remain under investigation. In this context, bioelectrical impedance analysis may represent a complementary tool, particularly when direct electrical parameters are interpreted alongside other functional and structural assessments.

The relationship between PA and NT-proBNP observed in our analysis is particularly noteworthy. NT-proBNP is a well-established marker of haemodynamic stress and prognosis in heart failure, yet its interpretation may be influenced by factors such as age and volume status. The independent association between lower PA and higher NT-proBNP concentrations suggests that BIA-derived markers capture dimensions of disease severity that are only partially overlapping with traditional cardiac parameters such as left ventricular ejection fraction. This observation is consistent with previous reports indicating that PA provides prognostic information beyond conventional clinical indices, although direct comparisons across studies remain challenging due to heterogeneity in patient populations, devices and impedance protocols [6,9,40,41,42,43].

Hydration-related parameters, especially total body water, showed an even stronger association with NT-proBNP and demonstrated good discriminatory capacity for identifying patients in the highest NT-proBNP quartile. This finding aligns with the central role of fluid overload in the pathophysiology of heart failure and highlights the potential utility of BIA for detecting subclinical congestion [25]. Similar applications of BIA have been extensively explored in other chronic conditions characterised by altered fluid distribution, most notably chronic kidney disease and dialysis populations [16,17]. In these settings, overhydration assessed by BIA has been consistently linked to worse functional status and increased mortality. Nevertheless, the mechanisms underlying fluid imbalance in heart failure differ substantially from those in renal disease, and extrapolation between these populations should be made with caution. While dialysis patients experience abrupt and externally modulated fluid shifts, patients with chronic HF typically exhibit more gradual and heterogeneous patterns of fluid accumulation, often accompanied by sarcopenia and adipose tissue redistribution [44,45].

Importantly, our results suggest that hydration and cellular health represent distinct but complementary dimensions of disease severity. While total body water was more strongly associated with NT-proBNP levels, PA showed a closer relationship with symptom burden, as reflected by NYHA class. This dissociation may have practical implications, as it indicates that bioimpedance analysis could help disentangle the relative contributions of congestion and nutritional or muscular impairment to patients’ clinical presentation. Such differentiation is particularly relevant in chronic HF, where dyspnoea and exercise intolerance are not exclusively driven by fluid overload and may persist despite apparently adequate decongestion [45,46].

From a pathophysiological perspective, the present findings support the concept that bioimpedance-derived electrical parameters capture complementary domains of chronic heart failure severity. Phase angle appears to reflect cellular integrity-related impairment, whereas hydration indices relate more closely to haemodynamic congestion. These dimensions are not fully overlapping and may contribute to multidimensional phenotyping rather than direct clinical decision-making.

### 4.3. Strengths, Limitations and Future Directions

The inclusion of a well-characterised cohort of patients with chronic HF assessed in a stable outpatient setting increases the clinical relevance of the findings. The simultaneous evaluation of symptom severity, biochemical markers and BIA-derived parameters allowed for an integrated analysis of functional, cardiac and body composition domains. Furthermore, multivariable modelling reduced the likelihood that the observed associations were driven solely by confounding clinical factors.

Several limitations should be acknowledged. The observational design precludes causal inference, and the sample size limits the ability to perform robust subgroup analyses, particularly according to sex, HF phenotype or degree of congestion. The very wide confidence interval observed for heart failure phenotype in the ordinal regression model also suggests limited precision of this estimate and likely reflects the relatively small sample size and subgroup imbalance. Therefore, the findings of the present study should be interpreted as exploratory and hypothesis-generating rather than as evidence supporting specific diagnostic thresholds or clinical decision rules. The proportion of women in the cohort was relatively low, which may restrict the generalisability of our findings. Additionally, the absence of longitudinal follow-up and hard clinical outcomes, such as mortality or hospitalisation, precluded assessment of the prognostic impact of BIA parameters beyond their cross-sectional associations. The study did not include direct haemodynamic or imaging-based markers of congestion, such as venous ultrasound scores or central venous pressure measurements. Therefore, the relationship between bioimpedance-derived hydration parameters and objective congestion indices could not be directly assessed. This question warrants dedicated investigation.

Future studies should evaluate whether modified or alternative criteria that account for fluid shifts yield more accurate and clinically relevant identification of malnutrition in heart failure.

Longitudinal studies are also needed to determine whether BIA-based assessment better predicts clinical outcomes and response to nutritional interventions compared with other techniques such as nutritional ultrasound or DXA. Finally, interventional studies integrating systematic nutritional screening and targeted nutritional support may help clarify whether improved identification of malnutrition translates into better functional and prognostic outcomes in chronic HF.

Although the present study was not designed to evaluate outcomes or intervention effects, the observed associations provide a rationale for future longitudinal and interventional research in this area.

## 5. Conclusions

In ambulatory patients with chronic heart failure, bioelectrical impedance-derived parameters reflecting cellular integrity and hydration status are independently associated with clinical and biochemical markers of disease severity. Direct electrical measurements such as phase angle may provide complementary information in the nutritional and clinical assessment of these patients, although hydration-related confounding should be carefully considered when interpreting body composition estimates. These findings support the potential role of bioelectrical impedance-derived electrical parameters as complementary markers in the multidimensional assessment of chronic heart failure.

## Figures and Tables

**Table 1:** General and cardiological characteristics.

		n = 115
Age (Years)	Mean ± SD	68.2 ± 12.6
Sex	n (%)	
Men		82 (71.3)
Women		33 (28.7)
Previous diabetes	n (%)	
No		80 (69.6)
Yes		35 (30.4)
Heart failure stage (ACC/AHA): B–C	n (%)	
B		65 (56.5)
C		50 (43.5)
Dyspnea grade	n (%)	
I		43 (37.4)
II		51 (44.3)
III		18 (15.7)
IV		3 (2.6)
Ejection fraction	mean ± SD	38.4 ± 10.8
NT-proBNP (pg/mL)	mean ± SD	3171 ± 5727

Abbreviations: SD = Standard Deviation.

**Table 2:** Body composition parameters (BIA) and Global Leadership Initiative on Malnutrition (GLIM) phenotypic criteria prevalence.

		All	Women	Men	*p* Value
		n = 115	n = 33	n = 82	
Body-mass index (kg/m^2^)	mean ± SD	29.6 ± 6.3	30.4 ± 6.2	29.4 ± 6.4	0.44
Low BMI (adjusted for age) *	n (%)	8 (7)	1 (3)	7 (8.6)	
Weight loss >5% in <6 months or >10% in >6 months *	n (%)	26 (22.8)	5 (15.2)	21 (25.9)	
Rz	mean ± SD	506 ± 96.3	556 ± 89.6	482.6 ± 88.9	<0.001
Xc	mean ± SD	40.5 ± 11.3	44.9 ± 10.9	38.7 ± 11	0.007
PA (°)	mean ± SD	4.6 ± 1.1	4.6 ± 1.1	4.6 ± 1.2	0.96
SPA	mean ± SD	−1.03 ± 1.11	−0.71 ± 1.05	−1.15 ± 1.11	0.067
TBW (L)	mean ± SD	41.4 ± 8.8	34.1 ± 5.8	44.3 ± 7.9	<0.001
TBW (%)	mean ± SD	51.6 ± 7.4	45.5 ± 6.3	54.2 ± 6.2	<0.001
ECW (%)	mean ± SD	21.6 ± 5.2	17.9 ± 2.8	23.1 ± 5.2	<0.001
BCM (kg)	mean ± SD	23.4 ± 6.7	19.8 ± 5.5	24.8 ± 6.6	<0.001
FFM (kg)	mean ± SD	53.2 ± 10.4	44.1 ± 8.4	56.8 ± 8.8	<0.001
FFMI (kg/m^2^)	mean ± SD	19.2 ± 2.9	17.5 ± 2.7	19.9 ± 2.7	<0.001
Below ESPEN cutoff points	n (%)	16 (14)	3 (9.1)	13 (16)	
FM (kg)	mean ± SD	28.2 ± 13.1	32.9 ± 13.8	26.3 ± 12.4	0.015
ASMM (kg)	mean ± SD	20.8 ± 5.1	17 ± 4.2	22.3 ± 4.6	<0.001
ASMMI (kg/m^2^)	mean ± SD	7.40 ± 1.68	6.71± 1.32	7.67 ± 1.60	<0.001
Below ESPEN cutoff points	n (%)	27 (23.7)	7 (21.2)	20 (24.7)	
Hydragram^®^, %	mean ± SD	76.6 ± 4.8	75.1 ± 3.5	77.2 ± 5.1	0.043
Nutrigram^®^, mg/24 h/m	mean ± SD	741 ± 213	633.4 ± 182.4	783.8 ± 210.5	<0.001
Presence of at least one GLIM phenotypic criterion	n (%)	45 (39.5)	12 (36.4)	33 (40.7)	

* According to GLIM criteria.

**Table 3:** Factors associated with dyspnoea severity (NYHA I–IV).

Variable	OR (95% CI)	*p* Value
Age (per year)	1.06 (1.01–1.11)	0.012
Sex (men vs. women)	2.09 (0.77–5.80)	0.151
Body mass index (kg/m^2^)	1.02 (0.99–1.05)	0.143
Left ventricular ejection fraction (%)	0.98 (0.94–1.01)	0.236
Heart failure type (diastolic vs. systolic)	4.34 (0.11–180.16)	0.404
Phase angle (°)	0.58 (0.35–0.93)	0.026
Total body water (L)	1.07 (1.02–1.13)	0.013

Model fit: McFadden R^2^ = 0.166; Likelihood ratio test χ^2^ = 34.2 (7 df), *p* < 0.001. Odds ratios represent the likelihood of being in a higher NYHA dyspnea class. Values < 1 indicate a protective association. Association between BIA-derived variables and NYHA class was assessed using ordinal logistic regression, assuming proportional odds across adjacent NYHA categories. The proportional odds assumption of the ordinal logistic regression models was formally assessed and showed no relevant violations.

**Table 4:** Multivariable linear regression analysis for log-transformed NT-proBNP.

Variable	β Coefficient (95% CI)	*p* Value
Age (per year)	0.01 (−0.02 to 0.03)	0.472
Sex (men vs. women)	0.46 (−0.10 to 1.02)	0.113
Body mass index (kg/m^2^)	0.00 (−0.01 to 0.02)	0.817
Heart failure type (diastolic vs. systolic)	−0.95 (−3.21 to 1.30)	0.405
Left ventricular ejection fraction (%)	−0.04 (−0.06 to −0.02)	<0.001
Phase angle (°)	−0.35 (−0.63 to −0.07)	0.014
Total body water (%)	0.08 (0.04 to 0.12)	<0.001

Regression coefficients represent the change in log-transformed NT-proBNP per unit increase in each predictor. Model performance: R^2^ = 0.415 Model diagnostics: Residuals were normally distributed and no heteroscedasticity or multicollinearity was detected (all VIF < 2).

**Table 5:** BIA-derived parameters associated with a high NT-proBNP phenotype.

Predictor	OR (95% CI)	*p* Value	McFadden R^2^	AUC
Phase angle (°)	0.33 (0.19–0.57)	<0.001	0.169	0.772
Total body water (%)	1.25 (1.14–1.38)	<0.001	0.261	0.824

Binary logistic regression analyses. Odds ratios represent the likelihood of belonging to the highest NT-proBNP quartile. AUC values were derived from receiver operating characteristic analyses.

## Data Availability

The data presented in this study are available on request from the corresponding authors due to privacy, legal and ethical reasons.

## Supplementary Materials

The following supporting information can be downloaded at https://www.mdpi.com/article/10.3390/jcm15062315/s1, Table S1: Correlations between bioimpedance-derived parameters and muscle ultra-sound/strength variables.

## Abbreviations

The following abbreviations are used in this manuscript:

AHA	American Heart Association
NYHA	New York Heart Association
ACC	American College of Cardiology
GLIM	Global Leadership Initiative on Malnutrition
ESPEN	The European Society for Clinical Nutrition and Metabolism
SGA	Subjective Global Assessment
NT-proBNP	N-terminal pro-B-type Natriuretic Peptide
HF	Heart Failure
EWGSOP	European Working Group on Sarcopenia in Older People
BIVA	Biolectrical Impedance Vector Analysis
Rz	Resistance normalised by height
Xc	Reactance normalised by height
PA	Phase Angle
SPA	Standardised Phase Angle
BIA	Bioelectrical Impedance Analysis
TBW	Total Body Water
ECW	Extracellular Water
BCM	Body Cell Mass
FFM	Fat-Free Mass
FFMI	Fat-Free Mass Index
FM	Fat Mass
SMM	Skeletal Muscle Mass
ASMM	Appendicular Skeletal Muscle Mass
BMI	Body Mass Index
MUAC	Mid Upper Arm Circumference
CC	Calf Circumference
SD	Standard Deviation
VIF	Variance Inflation Factor
DXA	Dual-energy X-ray Absorptiometry
