# Supplementary material for: Associations of Bioelectrical Impedance-Derived Phase Angle and Hydration Parameters with Clinical Severity in Ambulatory Chronic Heart Failure
Source: J Clin Med. 2026 Mar 18;15(6):2315. doi: 10.3390/jcm15062315 (PMC13026784; doi:10.3390/jcm15062315)
Supplement: Supplementary file 1 [file jcm-15-02315-s001.zip › jcm-4188356-supplementary.pdf]

Supplementary material

Supplementary Table S1. Correlations between bioimpedance-derived parameters and muscle ultrasound/strength variables

**Variable**	**Phase angle (°)**	**Total body water (%)**
Rectus femoris cross-sectional area	0.544***	−0.085
Rectus femoris thickness (Y-axis)	0.563***	−0.232*
Handgrip strength	0.424***	0.118

Values are Pearson correlation coefficients (r). $p < 0.05$; ** $p < 0.01$; *** $p < 0.001$. Muscle ultrasound and handgrip variables were analysed exclusively for convergent validity with bioimpedance-derived parameters and are therefore not descriptively reported.
